# Sorting by reversals, block interchanges, tandem duplications, and deletions

**DOI:** 10.1186/1471-2105-10-S1-S9

**Published:** 2009-01-30

**Authors:** Martin Bader

**Affiliations:** 1 Institute of Theoretical Computer Science, University of Ulm, 89069 Ulm, Germany

## Abstract

**Background:**

Finding sequences of evolutionary operations that transform one genome into another is a classic problem in comparative genomics. While most of the genome rearrangement algorithms assume that there is exactly one copy of each gene in both genomes, this does not reflect the biological reality very well – most of the studied genomes contain duplicated gene content, which has to be removed before applying those algorithms. However, dealing with unequal gene content is a very challenging task, and only few algorithms allow operations like duplications and deletions. Almost all of these algorithms restrict these operations to have a fixed size.

**Results:**

In this paper, we present a heuristic algorithm to sort an ancestral genome (with unique gene content) into a genome of a descendant (with arbitrary gene content) by reversals, block interchanges, tandem duplications, and deletions, where tandem duplications and deletions are of arbitrary size.

**Conclusion:**

Experimental results show that our algorithm finds sorting sequences that are close to an optimal sorting sequence when the ancestor and the descendant are closely related. The quality of the results decreases when the genomes get more diverged or the genome size increases. Nevertheless, the calculated distances give a good approximation of the true evolutionary distances.

## Background

During evolution, genomes are subject to genome rearrangements, which are large scale mutations that can alter the ordering and orientation (strandedness) of the genes on the chromosomes or even change the genome content by inserting, deleting, or duplicating genes. Because these events are rare compared to point mutations, they can give us valuable information about ancient events in the evolutionary history of organisms. For this reason, one is interested in the most "plausible" genome rearrangement scenario between two genomes. More precisely, given two genomes, one wants to find an optimal (shortest) sequence of rearrangements that transforms this genome into the other. In the classical approach, each gene has exactly one copy in each genome, and only operations that do not change the genome content are considered. These "classical operations" are nowadays a well-studied subject, where the most important operations are *reversals *(also called *inversions*), where a section of the genome is excised, reversed in orientation, and reinserted, and *transpositions*, where a section of the genome is excised and reinserted at a new position in the genome. While the problem of *Sorting by reversals *can be solved in polynomial time [1-3], and the reversal distance can be determined in linear time [4], the problem gets more complicated if one also considers transpositions, and there are only approximation algorithms known [5-7]. To simplify the existing algorithms, Yancopoulos et al. invented the *double cut and join *operator, which can simulate reversals and block interchanges (a more generalized form of a transposition), resulting in a simple and efficient algorithm [8].

However, restricting the genes to be unique in each genome does not reflect the biological reality very well, as in most genomes that have been studied, there are some genes that are present in two or more copies. This holds especially for the genomes of plants, and one of the most prominent genomes is the one of the flowering plant *Arabidopsis thaliana*, where large segments of the genome have been duplicated (see e.g. [9]). There are various evolutionary events that can change the content of the genome, like duplications of single genes, horizontal gene transfer, or tandem duplications. For a nice overview in the context of comparative genomics, see [10]. From an algorithmic point of view, the existence of duplicated genes complicates many existing algorithms, for example the problem of sorting arbitrary strings by reversals [11] and the problem of sorting by reversals and duplications [12] have been proven to be NP-hard. So far, most of the existing algorithms restrict duplications to have a fixed length [13], or simulate duplications by arbitrary insertions [14-16]. Even with these restrictions, it is hard to solve most of the problems exactly, and heuristics have to be used.

### Related work

While genome rearrangement problems without duplications is a well studied subject, considering genomes with duplicated genes is a rather new field of research. One of the first works on this topic was done by Sankoff [17], where the following problem was examined. Given two genomes with duplicated genes, identify in both genomes the "true exemplars" of each gene and remove all other genes, such that the rearrangement distance between these modified genomes is minimized. This approach minimizes the number of classical rearrangement operations, but not the one of duplications and deletions. In the work of El-Mabrouk [18], for a given genome with duplicated gene content, one searches for a hypothetical ancestor with unique gene content such that the reversal and duplication distance towards this ancestor is minimized. Bertrand et al. [13] developed an algorithm for the following problem. Given two genomes with duplicated gene content, find a hypothetical ancestor such that the sum of the reversal and duplication distance of both genomes to this ancestor is minimized. However, in this work, duplications are restricted to have the length of one marker, i.e. a duplication can only duplicate segments that are identical in the initial genomes. Therefore, this approach is disadvantageous if large segmental duplications happened during evolution. Fu et al. extended this approach to the greedy algorithm MSOAR for assigning orthologous genes, which works well in practice [12,19]. Other approaches [14-16] simulate duplications by arbitrary insertions. Recently, Yancopoulos and Friedberg provided a mathematical model of a genome rearrangement distance for genomes with unequal gene content [20], combining the DCJ operator [8] with arbitrary but length-weighted insertions and deletions. Another field of research is the "Genome halving problem", where a rearrangement scenario consists of a whole genome duplication followed by a series of classical rearrangement operations. It has been studied first for reversals and translocations [21,22] and recently has been extended to the double cut and join operator [23,24].

To the best of our knowledge, the only approach that creates a rearrangement scenario between two genomes, consisting of duplications of arbitrary length and classical genome rearrangements, is the one of Ozery-Flato and Shamir [25]. They use a greedy algorithm that starts with one genome and in each step applies the simplest and most evident operation that brings this genome closer to the target genome. If there is no evident operation, the algorithm aborts. Although this approach fails on complicated rearrangement scenarios, they were able to find rearrangement scenarios for more than 98% of the karyotypes in the "Mitelman database of chromosome aberrations in cancer" [26].

### Our contribution

In this paper, we will focus on the following problem. Given an ancestral genome with unique gene content and the genome of a descendant with arbitrary gene content, find the shortest sequence of reversals, block interchanges, tandem duplications, and deletions that transforms the ancient genome into the one of the descendant. In contrast to most of the previous works, tandem duplications and deletions can be of arbitrary length. We developed a lower bound for the distance, and a heuristic greedy algorithm based on this lower bound. The approach can be extended to also include general duplications and insertions of single elements, as described in Section "Discussion". Experimental results on simulated data show that our algorithm works well in practice.

## Results

### Preliminaries

A *genome π *= (*π*_1 _... *π*_*n*_) is a string over the alphabet {1, ..., *n*}, where each element may have a positive or negative orientation (indicated by $\vec{x}$ or $\overset{\leftarrow }{x}$). An *augmented genome *is a genome where the element *π*_0 _= $\vec{0}$ is added at the beginning and the element *π*_*n*+1 _= $\vec{n+1}$ is added at the end. As our algorithm works on augmented genomes, we will use the term "genome" as short hand for augmented genome. The genome $\left(\vec{0}\vec{1}\cdots \vec{n}\vec{n+1}\right)$ is called the *identity genome id*. The *multiplicity *of an element is the number of its occurrences (with arbitrary orientation) in *π*. Two consecutive elements *π*_*i *_*π*_*i*+1 _form an *adjacency *if *π*_*i *_= $\vec{x}$ and *π*_*i*+1 _= $\vec{x+1}$, or if *π*_*i *_= $\overset{\leftarrow }{x}$ and *π*_*i*+1 _= $\overset{\leftarrow }{x-1}$. Otherwise, they form a *breakpoint*. A *segment π*_*i *_... *π*_*j *_(with *i *≤ *j*) of a genome *π *is a consecutive sequence of elements in *π*, with *π*_*i *_as first element and *π*_*j *_as last element. A *genome rearrangement problem *is defined as follows. Given two genomes *π*' and *π *and a set of possible operations, where each operation is assigned a weight, find a sequence of minimum weight that transforms *π*' into *π*. This minimum weight will be denoted by *d*(*π*', *π*). In our algorithm, we will restrict the set of operations to *reversals, deletions, tandem duplications *(all of weight 1), and *block interchanges *(of weight 2), as defined in the next subsection. For simplification, we will also assume that *π*' = *id*, i.e. we search for a sequence of operations that transforms the identity genome into *π*, and we write *d*(*π*) as short hand for *d*(*id*, *π*).

### Operations

In our algorithm, we will restrict the set of operations to the following four types of operations. A *reversal rev*(*i*, *j*) (with 0 <*i *<*j *<*n *+ 1) is an operation that inverts the the order of the elements of the segment *π*_*i *_... *π*_*j*-1_. Additionally, the orientation of every element in the segment is flipped. A *block interchange bi*(*i*, *j*, *k*, *l*) (with 0 <*i *≤ *j *≤ *k *≤ *l *<*n *+ 1) is an operation that changes the positions of the segments *π*_*i *_... *π*_*j*-1 _and *π*_*k *_... *π*_*l*-1 _in *π*. A *tandem duplication td*(*i*, *j*) (with 0 <*i *<*j *<*n *+ 1) is an operation that adds a copy of the segment *πi *... *π*_*j*-1 _before the element *π*_*j*_. A *deletion del*(*i*, *j*) (with 0 <*i *<*j *<*n *+ 1) cuts the segment *π*_*i *_... *π*_*j*-1 _out of *π*.

We will use the double cut and join operator (short DCJ) to simulate reversals and transpositions. A *double cut and join DCJ*(*i*, *j*, *x*) (with 0 <*i *<*j *<*n *+ 1 and *x *∈ {+, -}) cuts the genome *π *before the elements *π*_*i *_and *π*_*j *_(i.e. we get the segments *π*_0 _... *π*_*i*-1_, *π*_*i *_... *π*_*j*-1_, and *π*_*j *_... *π*_*n*+1_), and rejoins the cut ends in two new pairs. If *x *= +, we rejoin such that the elements *π*_*i*-1 _and *π*_*j*-1 _as well as the elements *π*_*i *_and *π*_*j*-1 _become adjacent. This is equivalent to a the reversal of the segment *π*_*i *_... *π*_*j*-1_, i.e. *DCJ*(*i*, *j*, +) = *rev*(*i*, *j*). If *x *= -, we rejoin such that the elements *π*_*i*-1 _and *π*_*j *_as well as the elements *π*_*i *_and *π*_*j*-1 _become adjacent. This cuts the genome into the linear genome *π*_0 _... *π*_*i*-1 _*π*_*j *_... *π*_*n*+1 _and the circular genome ... *π*_*j*-2 _*π*_*j*-1 _*π*_*i *_*π*_*i*+1 _.... This circular genome can be absorbed by applying another DCJ with one cutting point in the linear genome and the other cutting point in the circular genome. Depending on how we rejoin, those two DCJs are equivalent to either two consecutive reversals or to one block interchange. Thus, we can reduce the set of operations to DCJs, tandem duplications, and deletions, provided that we demand that circular genomes must be absorbed in the next step.

### The breakpoint graph

**Figure 1:**
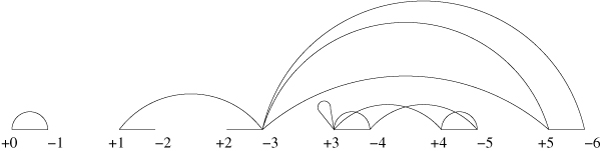
**The breakpoint graph**. The breakpoint graph of $\pi =(\vec{0}\vec{1}\vec{3}\overset{\leftarrow }{3}\overset{\leftarrow }{5}\vec{4}\overset{\leftarrow }{3}\overset{\leftarrow }{5}\overset{\leftarrow }{4}\overset{\leftarrow }{3}\vec{6})$. The edge (-3, +5) has a multiplicity of 2, all other edges have a multiplicity of 1. The edge (+3, +3) is a loop. The breakpoint graph consists of three components, the edge (+1, -3) is a 1-bridge, and the pair of edges (+3, +4), (-4, -5) is a 2-bridge.

Our main tool for visualization is the breakpoint graph. This graph has been introduced by Bafna and Pevzner to solve rearrangement problems on genomes without duplicated genes [27]. We extend this graph such that it can also be used for genomes with duplicated genes. The breakpoint graph of a genome *π *can be constructed as follows. First, we write the set of vertices {+0, -1, +1, -2, +2, ..., -*n*, +*n*, -(*n *+ 1)} from left to right on a straight line. Second, we add a *reality edge *(+*i*, -(*i *+ 1)) for each *i *∈ [0, *n*]. Third, we add a *desire edge *(*v*, *v'*) for each *i *∈ [0, *n*], where *v *= +*π*_*i *_if *π*_*i *_has a positive orientation, *v *= -*π*_*i *_otherwise, and *v' *= -*π*_*i*+1 _if *π*_*i*+1 _has a positive orientation, *v' *= +*π*_*i*+1 _otherwise. For better readability, we draw reality edges as straight lines and desire edges as arcs. For an example, see Fig. 1. In contrast to the original breakpoint graph, each vertex can be the endpoint of several desire edges. In fact, the number of desire edges connected to a vertex +*x *or -*x *is exactly the multiplicity of the element *x *in *π*. The *multiplicity *of an edge (*v*, *v'*) is the number of desire edges between *v *and *v'*. A desire edge (*v*, *v*) is called a *loop*. Let *S*(*π*) denote the number of vertices with a loop. Two vertices *v*, *v' *are in the same *componen*t of the graph if and only if there is a path (consisting of reality edges and desire edges) from v to *v'*. Let *C*(*π*) denote the number of components in the breakpoint graph of *π*. An edge is called a *1-bridge *if the removal of this edge increases *C*(*π*). A pair of edges is called a *2-bridge *if none of the edges is a 1-bridge and the removal of both edges increases *C*.

Note that two different genomes can have the same breakpoint graph, like e.g. $\left(\vec{0}\vec{1}\vec{2}\vec{1}\vec{3}\right)$ and $\left(\vec{0}\vec{1}\overset{\leftarrow }{2}\vec{1}\vec{3}\right)$. However, this will not cause problems in our algorithm, because we use the identity genome as ancestral genome, which has a unique breakpoint graph.

### A lower bound

Instead of searching for a sequence of operations *op*_1_, ..., *op*_*k *_that sorts *id *into *π*, one can also search for the inverse sequence $op_{k}^{-1},\cdots ,op_{1}^{-1}$ that sorts *π *into *id*. This is more convenient, as it is easier to track the changes in the breakpoint graph caused by the performed operations (remember that the reality edges are defined as the adjacencies in the identity genome). Thus, we only apply inverse operations, i.e. we sort a genome *π *into *id *by DCJs, inverse tandem duplications, and inverse deletions. Note that the inverse of a DCJ is still a DCJ, while an inverse deletion is an insertion. To keep our original problem in mind, we will use the term "inverse deletion" and not "insertion".

**Lemma 1**. *The breakpoint graph of the identity genome has n *+ 1 *components and no loops. No breakpoint graph has more components. If a breakpoint graph has n *+ 1 *components, it is the breakpoint graph of the identity genome if and only if it has no loops*.

*Proof*. The first statement is easy to verify. As each vertex is connected with another vertex by a reality edge, the maximum possible number of components in the breakpoint graph is *n *+ 1. If a genome is not the *id*, it must contain a breakpoint. The desire edge corresponding to this breakpoint either is a loop, or it connects two vertices that are not connected by a reality edge. In the latter case, the breakpoint graph contains a component with at least 4 vertices and therefore cannot have *n *+ 1 components.   □

We will now examine how an operation can change the number of components and loops.

#### DCJ

A DCJ cuts the genome at two positions, and rejoins the cut ends. This has the following effect on the breakpoint graph. Two desire edges (*u*, *v*) and (*w*, *x*) are removed, and w.l.o.g. the desire edges (*u*, *w*) and (*v*, *x*) are added to the breakpoint graph. This can increase *C *by at most 1. If one of the removed edges is a loop, all three vertices are in the same component after the operation, i.e. *C *will not be increased by this operation. As a DCJ removes only two edges, *S *can be decreased by at most 2.

#### Inverse tandem duplication

An inverse tandem duplication deletes the following desire edges. (a) Edges that are inside the duplicated segment. All these edges have a multiplicity ≥ 2, thus deleting these edges neither changes *C *nor *S*. (b) The edge between the last node of the segment and the first node of the copy. This can increase *C *by 1, or decrease *S *by 1 (but not both).

#### Inverse deletion

An inverse deletion splits the genome at one position and adds arbitrary elements. In the breakpoint graph, one desire edge is removed and several desire edges are added. Therefore, a deletion can increase *C *by at most 1 or decrease *S *by at most 1. As *C *can only be decreased if the removed edge is a 1-bridge, an inverse deletion cannot increase *C *and decrease *S*.

**Theorem 1**. *A lower bound lb*(*π*) *of the distance d*(*π*) *is*

$$d(\pi )\geq lb(\pi )=n+1-C(\pi )+\sum_{Components}\left\lceil \frac{S_{i}}{2}\right\rceil$$

*where S*_*i *_*is the number of vertices with a loop in component C*_*i*_.

*Proof*. Operations that increase *C*(*π*) by 1 or decrease *S*(*π*) by 1 decrease the lower bound at most by 1. For an operation that remove two loops, there are two cases. (a) It acts on two loops of the same component *C*_*i*_. This decreases *S*_*i *_by 2 and the lower bound is decreased by 1. (b) It acts on two loops in two components *C*_*i *_and *C*_*j*_. This can decrease two of the summands by 1, but the components *C*_*i *_and *C*_*j *_are merged and *C *is decreased by 1, thus the lower bound is decreased by at most 1.   □

### The algorithm

The algorithm uses a greedy strategy to sort the genome. In each step, it searches for operations that decrease the lower bound, i.e. we search for operations that increase *C *or decrease *S*, and check their effect on the lower bound. If there is no such operation, we will use additional heuristics to search for small sequences of operations that bring us closer to our goal. The main idea behind these heuristics is to reduce the number of missing elements and duplicates and to create adjacencies.

### Operations that decrease the lower bound

As a DCJ removes two desire edges and rejoins the endpoints with two new desire edges, it can only increase *C *if the removed desire edges are a 2-bridge, or two 1-bridges in the same component. If the DCJ rejoins the endpoints such that we get a linear and a circular genome, we need a lookahead to search for another DCJ that absorbs this DCJ. Those two DCJs are directly merged into two reversals or one block interchange with a weight of 2. Inverse tandem duplications can only remove one desire edge with a multiplicity of 1 (the one between the duplicated segments), thus an inverse tandem duplication can increase *C *only if this edge is a 1-bridge. Additionally, one has to check whether the segments on both sides of the cutting point are identical. Inverse deletions just remove one desire edge, thus also an inverse deletion can increase *C *only if the removed edge is a 1-bridge. Additionally, one has to check whether there is a segment that can be inserted such that no desire edge in the inserted segment merges two components. Although there is such a segment in most cases, practical tests have shown that it is better to only insert segments that have no breakpoints, i.e. we perform only an inverse deletion if the breakpoint is of the form $\vec{x}\vec{y}$ or $\overset{\leftarrow }{y}\overset{\leftarrow }{x}$ with *x *<*y*. In summary, the main task in finding operations that increase *C *is to find 1-bridges and 2-bridges in the breakpoint graph, which can be done very efficiently by following the algorithm devised in [28].

Finding operations that decrease *S *is rather straightforward, as we just have to scan the breakpoint graph for loops with a multiplicity of 1 and find the corresponding position in the genome. An operation that decreases *S *can be an inverse tandem duplication or an inverse deletion that removes this loop, or a DCJ that removes two loops with a multiplicity of 1, or a DCJ on a loop and another desire edge of the same component.

### Heuristics for the remaining cases

If there is no operation that decreases the lower bound, one heuristic would be to decrease the number of duplicated elements without increasing the lower bound. If there are two consecutive copies of the same segment, we can remove one of them by an inverse tandem duplication. As an inverse tandem duplication only removes desire edges, it can never increase the lower bound. This is different in the general case of an inverse duplication, where the duplicated segments are separated by a non-empty segment in the genome. In this case, the removal of one of these segments (which can be simulated by a block interchange and an inverse tandem duplication) creates a new desire edge between the last element before the removed segment and the first element after the removed segment. If the corresponding vertices in the breakpoint graph are in different components, or if they are identical and the new desire edge would increase ⌊*S*_*i*_/2⌋ of this component, the operation would increase the lower bound, i.e. we cannot easily provide a sequence of operations that removes one of the duplicated segments and does not increase the lower bound. However, the situation is different if we have at least three copies of the segment.

**Lemma 2**. *If there are three identical copies of a segment that are maximal (i.e. they cannot be extended in any direction such that still all three copies are identical), then there exists a sequence of operations that removes two of these copies and does not increase the lower bound*.

**Figure 2:**
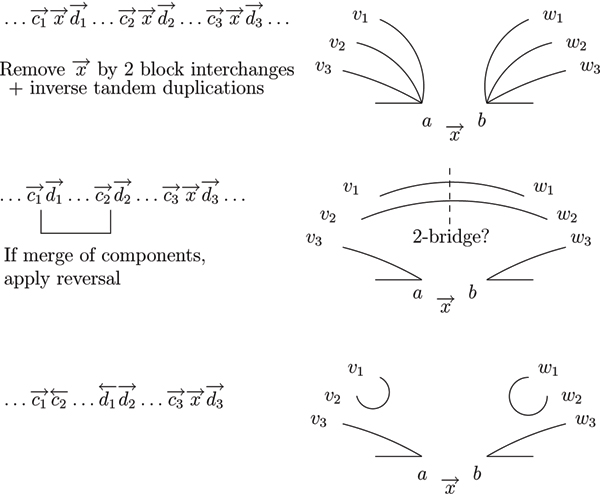
**Sequences for elements with multiplicity = 3**. An example for a sequence if an element × has a multiplicity ≥ 3. First, we remove two of the copies of *x *by two block interchanges and inverse tandem duplications. If this merges two components, the component can be cut by an additional reversal. For other orientations of the element *x*, similar sequences can be applied.

*Proof*. Let *a *be the vertex corresponding to the leftmost element of the segment, and let *b *be the vertex corresponding to the rightmost vertex of the segment. There are reality edges (*v*_1_, *a*) and (*b*, *w*_1_), (v_2_, *a*) and (*b*, *w*_2_), and (*v*_3_, *a*) and (*b*, *w*_3_) (from the elements enclosing the first, second, and third copy of the segment). Because the segment is maximal, we can assume w.l.o.g. that *w*_1 _≠ *w*_2_. As *v*_1_, *v*_2_, and *v*_3 _are all adjacent to *a*, they must be in the same component, as well as *w*_1_, *w*_2_, and *w*_3_. By deleting the first two segments, we remove the desire edges (*v*_1_, *a*), (*b*, *w*_1_), (*v*_2_, *a*), and (*b*, *w*_2_), and get the new desire edges (*v*_1_, *w*_1_) and (*v*_2_, *w*_2_). If this merges two components, the new desire edges are a 2-bridge, and we can apply a DCJ that replaces them by the desire edges (*v*_1_, *v*_2_) and (*w*_1_, *w*_2_). If *v*_1 _= *v*_2 _this can create a new loop. This loop can be removed by another DCJ between the edges (*v*_1_, *v*_2_) and (*v*_3_, *a*) (note that *v*_3 _≠ *v*_1 _because the segments are maximal, and *v*_1 _≠ *a *because otherwise the loop was already there before the operation). In fact, the operations of the sequence can be arranged such that all DCJs are reversals, so we do not have to find appropriate follow-ups. An illustration of the sequence is depicted in Fig. 2.   □

We will now examine what we can do with elements with a multiplicity of at most 2. A first strategy would be to create adjacencies wherever this is possible without creating loops (note that creating adjacencies cannot decrease *C*). As a precondition, there must be a reality edge (*a*, *b*) and the desire edges (*a*, *c*) and (*b*, *d*) with *c *≠ *d*.

**Figure 3:**
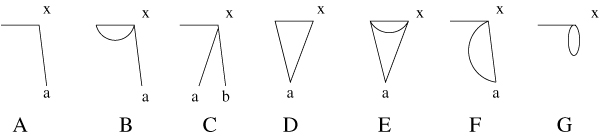
**The remaining cases**. The different configurations in which each vertex is adjacent to at most two desire edges, and no adjacencies can be created without creating a loop. In all cases, the picture shows a reality edge (horizontal line) and its adjacent desire edges (all other lines).

If there are no further adjacencies to create, and all elements have a multiplicity of at most 2, all the possible cases for a reality edge and its adjacent desire edges are depicted in Fig. 3.

**Lemma 3**. *If all elements in π have a multiplicity ≤ *2*, and there is no DCJ that creates an adjacency without creating a loop, then there is a reality edge with adjacent desire edges corresponding to Case A, B, or C in Fig*. 3*. For these cases, there is an operation or a sequence of operations that removes this configuration*.

*Proof*. If a reality edge and its adjacent desire edges correspond to Case D or E, then the reality edge starting at vertex *a *must correspond to Case C (this follows from the preconditions). Now, let us assume that all reality edges correspond to one of the Cases F and G. The elements adjacent to a reality edge of these cases occur either twice in the genome, or they do not occur at all. As we work with augmented genomes, there must be at least two elements that occur exactly once in the genome and have a breakpoint, otherwise the genome is the *id*. This is a contradiction to our assumption, therefore there must be at least one reality edge corresponding to Cases A, B, or C. We will now provide sequences for these cases.

#### Case A

The genome is of the form $\pi =(\ldots \vec{a}\vec{x}\ldots )$, the element *x *- 1 is missing. Let *y *be the largest element <*x *that is not missing. We apply an inverse deletion of the elements $\vec{y+1}$ to $\vec{x-1}$ between $\vec{a}$ and $\vec{x}$, i.e. *π *becomes $\left(\ldots \vec{a}\vec{y+1}\ldots \vec{x-1}\vec{x}\ldots \right)$. The desire edge (+*a*, -*x*) is removed, the inserted desire edges are the edge (+*a*, -(*y *+ 1)) and some adjacencies. The reality edge (+(*x *- 1), -*x*) is split from the component, the edge (+*a*, -(*y *+ 1)) may merge two components, so the overall number of components cannot be decreased. As the element *y *+ 1 was not present in the original genome, the edge (+*a*, -(*y *+ 1)) cannot be a loop.

#### Case B

*x *is in a duplicated segment, w.l.o.g. the segment is left-maximal. We extend it to the right until it is also right-maximal. Nevertheless, we will denote the leftmost vertex of the duplicated segment by -*x *and the rightmost vertex by +*x*, i.e. $\pi =(\ldots \vec{x-1}\vec{x}\vec{b}\ldots \vec{a}\vec{x}\vec{c}\ldots )$ (the copies of *x *can also have negative orientation). As the segment is right-maximal, -*b *≠ -*c *or the segments have different orientation and touch each other, i.e. $\pi =(\ldots \vec{x-1}\vec{x}\overset{\leftarrow }{x}\overset{\leftarrow }{a}\ldots )$. In the first case, we remove the copy of *x *that is not adjacent to *x *- 1, i.e. we remove the desire edges (+*a*, -*x*) and (+*x*, -*c*), and create the new desire edge (+*a*, -*c*). If +*a *= -*c*, the loop can be removed by a DCJ on this edge and the edge (+*x*, -*b*). In the second case, we remove the copy of *x *that is not adjacent to *x *- 1, i.e. we remove the loop and the desire edge (+*a*, -*x*), and we create the desire edge (+*x*, +*a*). In both cases, the desire edge (+(*x *- 1), -*x*) is split from the component, and adding one new desire edge can merge only two components, so the overall number of components does not decrease. Additionally, also *S *cannot increase.

#### Case C

The genome is of the form $\pi =(\ldots \vec{a}\vec{x}\vec{c}\ldots \vec{b}\vec{x}\vec{d}\ldots )$. We remove the second copy of *x*. This removes the desire edges (+*b*, -*x*) and (+*x*, -*d*) and adds the desire edge (+*b*, -*d*). If this has merged two components, then (+*a*, -*x*) and (+*b*, -*d*) are 1-bridges with disjoint endpoints (remember that there is no desire edge from vertex +(*x *- 1)), so a DCJ on these two edges splits the component again. If +*b *= -*d*, we have a loop, so we will not apply this sequence. Instead, we use the symmetrical case in which we remove the first copy of *x*. If both +*b *= -*d *and +*a *= -*c*, we can remove the loop (+*b*, -*d*) by applying a DCJ on it and the desire edge (+a, -*x*). Note that there is the possibility that the first DCJ creates a circular genome that cannot be absorbed in the next step. In this case, we can apply the sequence for Case A twice, i.e. we add the same elements before both copies of *x*.   □

### Completeness of the algorithm

**Figure 4:**
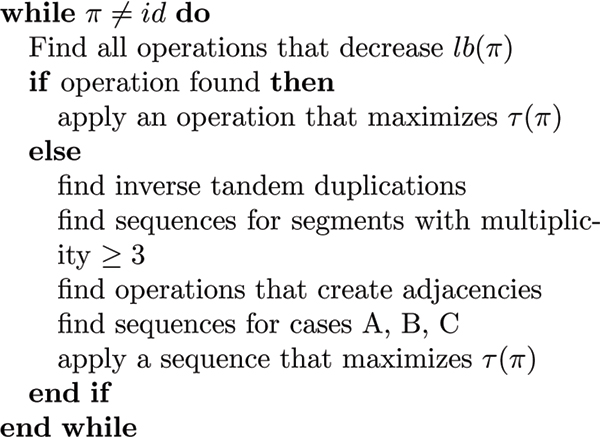
**The algorithm**. The algorithm in pseudocode.

Whenever the algorithm cannot apply an operation that decreases the lower bound, it searches for sequences that remove duplicated segments, for operations that create adjacencies, and for sequences according to the cases A to C in the previous subsection. Then, one of these sequences is selected and applied to the genome. The pseudocode of the algorithm can be seen in Fig. 4.

To prove the completeness of the algorithm, we need the following lemma.

**Lemma 4**. *Let m*(*π*) *be the number of missing elements in π, let r*(*π*) *the number of elements that have to be removed from π (i.e. r*(*π*) = |*π*| + *m*(*π*) - (*n *+ 2)*), and let a*(*π*) *be the number of adjacencies in π. Then, τ*(*π*): = *a*(*π*) - 2·(*m*(*π*) + *r*(*π*)) *is maximal if and only if π = id*.

Proof. Let *π *be a genome such that *τ*(*π*) is maximal. If *m*(*π*) > 0 we could transform *π *by adding all missing elements without increasing *r*(*π*) or decreasing *a*(*π*). This would be a contradiction to the fact that *τ*(*π*) is maximal, therefore *m*(*π*) = 0. Now, let us assume that *r*(*π*) > 0, and let *x *be the smallest element that is duplicated. As *x *- 1 has a multiplicity of 1, there is at least one copy of *x *that has a breakpoint. Removing this copy decreases *r*(*π*) by 1 and *a*(*π*) by at most 1, while *m*(*π*) remains unchanged. This would increase *τ*(*π*), and lead to a contradiction. Therefore, *π *is the genome without duplicated or missing elements with the maximum number of adjacencies, i.e. *π *= *id*.   □

We are now ready to prove the following theorem.

**Theorem 2**. *The algorithm terminates after a finite number of steps. When the algorithm terminates, the genome π is transformed into id*.

*Proof*. As none of the operations and sequences of operations discussed above increases the lower bound, and the lower bound is minimized for *id*, only a finite number of operations that decrease the lower bound can be applied. As we have shown in the last subsections, the algorithm always finds a sequence of operations as long as *π *≠ *id*. Table 1 shows the changes of *τ*(*π*) when applying these sequences. As all sequences increase *τ*(*π*), only a finite number of those sequences can be applied between two operations that decrease the lower bound. Therefore, the algorithm must terminate, and *π *is transformed into *id*.   □

**Table 1:** Estimating Δ*τ*. Changes of *m*(*π*), r(*π*), *a*(*π*), and *τ *(*π*) by applying the different sequences of operations described in this section. Case C' is the case where we cannot solve Case C directly and have to apply the sequence for Case A twice.*l *denotes the length of inserted or removed segments. Note that for all sequences, Δ*τ *> 0.

Sequence	Δ*m*(*π*)	Δ*r*(*π*)	Δ*a*(*π*)	Δ*τ*(*π*)
Inverse Tandem Duplication	0	-*l*	≥ -*l*	≥ *l*
Segments with multiplicity ≥ 3	0	-2*l*	≥ -2*l *- 1	≥ 2*l *- 1
Creating adjacencies	0	0	≥ 1	≥ 1
Case A	-*l*	0	*l*	2*l*
Case B	0	-*l*	≥ -*l*	≥ *l*
Case C	0	*l*	≥ -*l*	≥ *l*
Case C'	-*l*	*l*	2*l*	2*l*

### Testing

We used simulated data to assess the performance of our algorithm. We generated test cases by creating the identity genome *id *of size *n *and applying random sequences of *αn *operations for different values of *n *and *α *(namely *n *∈ {20, 50, 80, 100} and *α *from 0.1 to 1 in steps of 0.1). For each value of *n *and *α*, we created 10 test cases. The operations of the sequences are independently distributed, with tandem duplications and deletions having a probability of $\frac{1}{3}$, reversals having a probability of $\frac{2}{9}$, and block interchanges having a probability of $\frac{1}{9}$ (thus the expected numbers of DCJs, tandem duplications, and deletions are equal). Once the type of an operation was determined, the operation was selected uniformly distributed among all operations of this type. As long deletions can cancel the effects of previous operations, deletions were restricted to have a length of at most 0.1 times the current genome length. To keep the size of the genome approximately constant, also tandem duplications were restricted to have a length of at most 0.1 times the current genome length.

**Figure 5:**
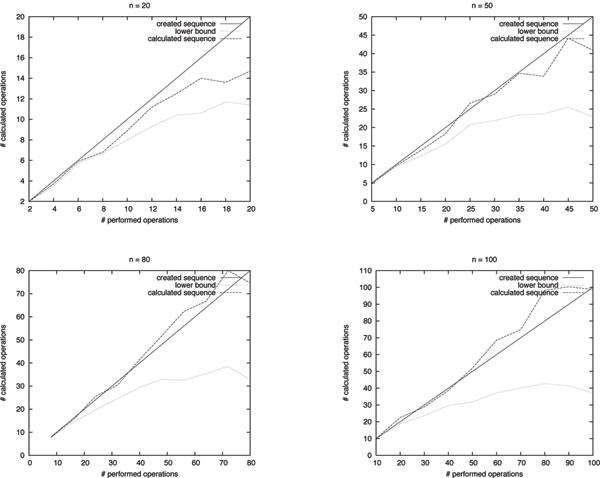
**Performance**. Performance of our algorithm on simulated data. In each diagram, the x-axis corresponds to the sequence weight used to obtain the test case, while the y-axis corresponds to the weight of the reconstructed sequence. Each value is the average of 10 created test cases.

**Figure 6:**
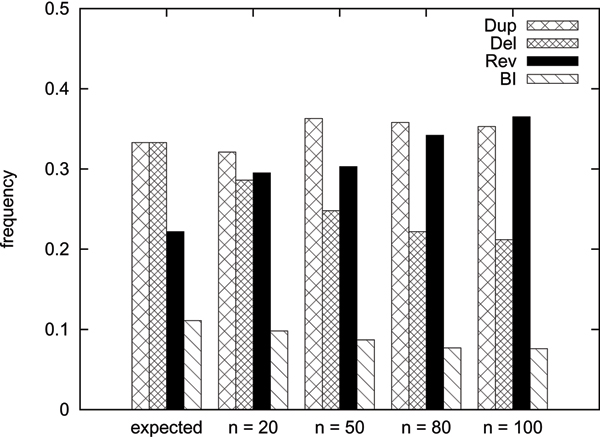
**Frequencies of the operations**. The relative frequencies of the different operations.

We then calculated the lower bounds of the test cases, and used our algorithm to reconstruct the sequence of operations. The results of these experiments can be seen in Fig. 5. On average, our algorithm finds good sequences (mostly with less operations than used to create the test case) as long as the lower bound is close to the number of operations used to create the test case. As this coherence lessens for increasing values of *n *and *α*, the length of the calculated sequences increases. However, even for higher values of *n *and *α*, the calculated distances are still a good approximation for the original distance. If one examines the frequency of the different types of operations, the number of performed duplications and block interchanges approximately fits the expected values. The algorithm tends to overestimate the number of reversals and underestimate the number of deletions, especially for higher values of *n*. For details, see Fig. 6.

## Discussion

In the following, we will discuss how the set of operations could be extended.

### Duplications

While our algorithm only considers tandem duplications, one might also be interested in including arbitrary duplications. This would be rather easy if tandem duplications have weight 1, and all other duplications have weight 2, since the general case of a duplication can change the lower bound by at most 2. However, weighting all duplications equally is a more complicated subject. If all duplications have weight 2, this could be disadvantageous in detecting tandem duplications, as an inverse tandem duplication can decrease the lower bound by at most 1. On the other hand, if all duplications have weight 1, duplications would be favored over DCJs. This could lead to sequences of many small duplications, instead of first merging the segments and then just applying one big duplication.

### Insertions

Insertions of single elements could be easily included in our algorithm, because the inverse of this operation decreases the lower bound by at most 1. Insertions of arbitrary length are more complicated. On the other hand, allowing insertions of arbitrary length are neither biologically meaningful nor do they make sense in combination with arbitrary deletions, because one could solve every sorting problem by just one deletion and one insertion step. Thus, further research in including insertions should also include a reasonable length depending weighting of the insertions.

## Conclusion

We presented an algorithm that works well for smaller genomes and distances. Although our results are promising, this algorithm should be seen as a first step in handling duplications of arbitrary length. Further research could improve the algorithm itself by finding closer lower bounds and better heuristics, or extend the algorithm such that it considers more different operations (as described in Section "Discussion") and can also handle multichromosomal genomes.

## Competing interests

The author declares that they have no competing interests.

## Authors' contributions

MB designed the algorithm, implemented it, performed the tests, and drafted this manuscript.
